# Model-Based Biomechanical Exoskeleton Concept Optimization for a Representative Lifting Task in Logistics

**DOI:** 10.3390/ijerph192315533

**Published:** 2022-11-23

**Authors:** Jonas Schiebl, Mark Tröster, Wiem Idoudi, Elena Gneiting, Leon Spies, Christophe Maufroy, Urs Schneider, Thomas Bauernhansl

**Affiliations:** 1 Fraunhofer Institute for Manufacturing Engineering and Automation IPA, 70569 Stuttgart, Germany; 2 Institute of Industrial Manufacturing and Management IFF, University of Stuttgart, 70569 Stuttgart, Germany

**Keywords:** logistics, ergonomics, manual work, exoskeleton, assistive systems, musculoskeletal modeling, multibody simulation, AnyBody Modeling System, activities above shoulder height, heavy lifting

## Abstract

Occupational exoskeletons are a promising solution to prevent work-related musculoskeletal disorders (WMSDs). However, there are no established systems that support heavy lifting to shoulder height. Thus, this work presents a model-based analysis of heavy lifting activities and subsequent exoskeleton concept optimization. Six motion sequences were captured in the laboratory for three subjects and analyzed in multibody simulations with respect to muscle activities (MAs) and joint forces (JFs). The most strenuous sequence was selected and utilized in further simulations of a human model connected to 32 exoskeleton concept variants. Six simulated concepts were compared concerning occurring JFs and MAs as well as interaction loads in the exoskeleton arm interfaces. Symmetric uplifting of a 21 kg box from hip to shoulder height was identified as the most strenuous motion sequence with highly loaded arms, shoulders, and back. Six concept variants reduced mean JFs (spine: >70%, glenohumeral joint: >69%) and MAs (back: >63%, shoulder: >59% in five concepts). Parasitic loads in the arm bracing varied strongly among variants. An exoskeleton design was identified that effectively supports heavy lifting, combining high musculoskeletal relief and low parasitic loads. The applied workflow can help developers in the optimization of exoskeletons.

## 1. Introduction

### 1.1. Musculoskeletal Disorders and Exoskeletons

Musculoskeletal disorders (MSD) are responsible for 60% of all reported work-related health problems in the European Union (EU) [1]. Among those, pain in the back (46% in 2015) and muscles of the shoulders, neck, and upper limbs (43% in 2015) are the most commonly reported issues [1]. Significant physical risk factors include working in awkward postures, moving or carrying heavy objects, and repetitive movements of the upper limbs [1]. In order to prevent MSDs, ergonomic working conditions should be improved by applying ergonomic design of working environments and training or instruction measures in a second step [2].

However, in constantly changing work environment requirements, organizational measures could be insufficient or inapplicable, for instance, when the work (e.g., in agriculture, construction, and logistics) is not linked to defined locations [3]. In those cases, occupational exoskeletons could relieve the worker [3]. Those wearable mechanical structures aim at reducing the physical load of workers by applying supportive forces or torques using passive (e.g., springs) or active actuators (e.g., electromechanical drives) [4]. In the last decades, various potential exoskeleton solutions for different industries were proposed, supporting either the lower, upper, or the whole body [5,6,7,8,9,10,11]. Those include primarily passive (e.g., Skelex 360-XFR [12], Ekso Evo [13], Ottobock Shoulder [14], MATE-XT [15], BESK G [16], Shiva Exo [17], Airframe [18], V3 ShoulderX [19]) but recently also a few (semi-)active (AGADEXO Shoulder [20], Shoulder exoskeleton S700 [21]) systems to support the back and shoulder during (repetitive) overhead work. Furthermore, there are systems to support the back during forward bending and the handling and lifting of heavy objects from the ground (e.g., Ottobock Back [22], HAL Lumbar Type for Labor Support [23], Cray X exoskeleton [24], Muscle Suit [25], Flex Lift [26], Laevo V2.5 [27], ExoBack [28], V3 BackX [29]).

However, there are other working conditions related to MSDs for which existing commercial solutions may not be helpful. Lifting and carrying heavy loads is associated with back pain and health conditions of the wrist, upper arms, and shoulders [30]. In addition, repeated heavy lifting to or above shoulder height was linked to neck and shoulder pain [31,32] and injuries [33]. It is yet unclear if and how shoulder-support exoskeletons optimized for overhead work (high shoulder flexion angles) can help during carrying and especially heavy lifting tasks from hip to or above shoulder height. There are indications that some systems could reduce muscle activities (MAs) during lifting the upper arm to shoulder level to a certain degree [34,35]. However, current systems provide only relatively low support torques of 3–6 Nm [36], which is why their capability to support heavy lifting effectively is questionable.

Furthermore, while exoskeletons have the potential to reduce muscular demands [4], their capability to prevent MSDs is not yet conclusive [3,4]. The redistribution of stress from one body part to another could potentially introduce new health risks, and exoskeletons could alter joint stability or user kinematics [3]. Hence, it is crucial to gain a better understanding of the biomechanical effects resulting from the use of exoskeletons. Therefore, simulation-based approaches utilizing multibody simulation software were used to analyze and optimize the effects of exoskeletons on the biomechanics of the human body during different tasks or loadings [37,38,39,40]. Compared with electromyography (EMG) and other physiological sensor-based evaluation methods, musculoskeletal models offer complementary holistic and in-detail insights into biomechanical interaction characteristics, such as joint reactions and grouped or single MAs [41,42]. Furthermore, they offer the opportunity to approximate metabolic cost [43,44], which is an interesting performance indicator for occupational exoskeletons, especially for dynamic manual material handling [36]. Those biomechanical outputs can be generated virtually for various motion sequences and exoskeleton design configurations [42,45].

In this context, Tröster et al. proposed a musculoskeletal-model-based exoskeleton design workflow in which real motion data of pulling and pushing tasks were captured, and body stresses were subsequently simulated. Analysis of the resulting body stresses helped to design and optimize an exoskeleton for manual pushing and pulling activities and investigate its effect on MAs and joint loading. However, the effect of the exoskeleton on the physical human–exoskeleton interface was only estimated with respect to overall interaction forces [46].

Parts of this workflow were applied to a preselected heavy lifting task, observed in the logistics work of the Bundeswehr (Armed Forces of Germany), which was then investigated concerning MAs and joint forces (JFs). Different support torques and forces were implemented in the model to compare abstract conceptual exoskeleton design approaches according to their musculoskeletal relief potential (hereinafter referred to as biomechanical effectiveness) [47].

Furthermore, requirements for exoskeletons designated for the use in machine maintenance and logistics in the Bundeswehr (Armed Forces of Germany) were gathered, including environmental requirements, available infrastructure, user information, safety, usability, and performance requirements [48]. In addition, the system to be developed needed to support overhead tasks and heavy lifting at shoulder height [48]. Based on the findings of [47,48], various exoskeleton architectures were researched and conceived. Finally, a concept was selected that met the requirements best and seemed promising regarding its musculoskeletal relief potential. In the present work the concept is further developed and evaluated, using a simulation-based approach.

### 1.2. Objective

Despite the connection between repeated lifting to or above shoulder level and MSDs [32,33], most commercially available shoulder exoskeletons are optimized to support overhead work. Hence, our work aims at designing a biomechanically effective exoskeleton to support workers in carrying and especially heavy lifting to or above shoulder height. In the first stage, we focus on the development of the mechanical design of the exoskeleton based on the previously selected rough concept.

To account for a biomechanically favorable interaction between the user and exoskeleton, we use a simulation-based workflow based on the one of Tröster et al. [46], extend it, and apply it to a heavy lifting task. Lifting motion sequences occurring during military logistics work scenarios are analyzed with respect to MAs and JFs in order to select the most strenuous sequence, which was already investigated in [47] on a more superficial level. For the selected task, the preselected mechanical exoskeleton CAD (computer-aided design) concept is subsequently presented and investigated regarding its biomechanical effectiveness using multibody simulations. Multiple variants of the said concept are simulated and analyzed with respect to carefully chosen biomechanical parameters, including MAs and JFs, as similarly described in [46]. In addition, forces and torques at the physical human–exoskeleton interface are investigated in order to make a first estimation of possible user discomfort due to interaction kinetics. Finally, a concept variant is chosen that shows good musculoskeletal relief combined with advantageous kinetics in the physical human–exoskeleton interfaces.

Hereby we hope to identify a suitable mechanical exoskeleton concept for heavy lifting from a biomechanical point of view and critically examine the potential of the applied workflow for optimizing mechanical exoskeleton models.

## 2. Methods

### 2.1. Workflow Overview

The proposed workflow is divided into two main parts. In the first part a subject motion is recorded via motion capture (Section 2.2 and Section 2.3). The motion data is then used to move a human model in a multibody simulation (kinematic analysis) and subsequently calculate resulting forces in an inverse dynamic analysis (Section 2.4). This is repeated for several subjects and motion sequences and analyzed with respect to selected biomechanical stress criteria. Finally, the most strenuous motion sequence is selected (Section 2.5). In the second part of the workflow, this motion sequence is simulated in combination with a virtually interfaced exoskeleton model (Section 2.6 and Section 2.7), hereby again executing kinematic and inverse dynamic analysis. This is repeated for multiple exoskeleton concept variants, and biomechanical stress criteria are analyzed in order to identify the concept with the highest musculoskeletal relief potential (Section 2.8.). Note that a more superficial analysis of one motion sequence (T5) and, consequently, also some workflow information from Section 2.2, Section 2.3, Section 2.4 and Section 2.5 were reported before [47]. However, for better readability, they are also included here in greater detail.

### 2.2. Test Subjects and Experimental Setup

For the biomechanical measurements in the laboratory, three male test subjects were recruited (age in years: 35 (±9), body height in cm: 181 (±9), body weight in kg: 74 (±5)). The subjects were informed in advance about potential risks during the manual handling procedures and gave their formal consent. 

**Figure 1:**
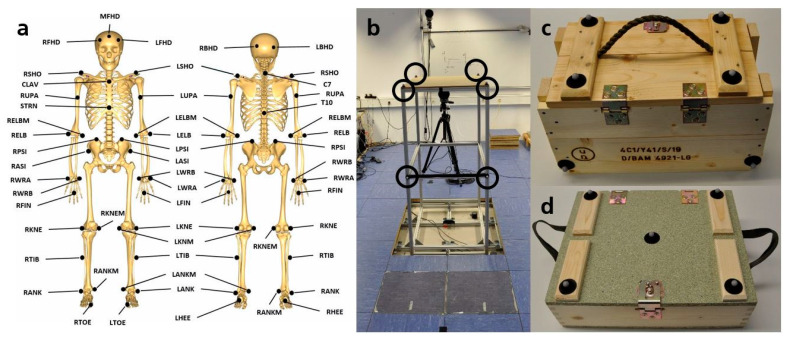
(**a**) Subject marker positions for optical motion markers. (**b**) Replicated loading platform with attached markers, highlighted with black circles. (**c**) Box A with attached markers and handle on top. (**d**) Box B with attached markers and two lateral handles.

The analyzed manual handling tasks were based on military logistics applications, including carrying heavy boxes (21 kg) and loading them on a truck, whereby the boxes had to be placed approximately at shoulder height. To replicate the manual loading procedure, a loading platform was built out of aluminum profiles (height: 146 cm, see Figure 1b). Two typical military logistics wood boxes were used (box A (length: 45 cm, width: 25 cm, height: 27 cm) and box B (length: 44 cm, width: 26 cm, height: 14 cm), see Figure 1c,d). To adjust the weight of the boxes, sandbags were placed inside.

In order to capture motions during the performed manual tasks, a marker-based optical system (Qualisys, Göteborg, Sweden) with 15 cameras (nine Oqus 700+ and six Oqus 400, Göteborg, Sweden) and the corresponding software analysis system (Qualisys Track Manager 2019.2, Qualisys, Göteborg, Sweden) were used. The cameras were calibrated in advance of the measurement procedure. A synchronized video camera (Oqus 210c, Qualisys, Göteborg, Sweden) captured the manual handling procedures. The motion data was recorded with a frequency of 100 Hz. To detect each human body segment´s positions, 43 markers were placed on each test subject according to the standard motion capture framework [49] of the AMMR (AnyBody Managed Model Repository) (see Figure 1a). Additional markers were placed on the loading platform and the boxes to accurately detect their motion (see Figure 1b–d). To prevent foot injuries, the test subjects had to wear safety shoes (Millenium Protect, Gaston Mille, Courthézon, France).

### 2.3. Experimental Procedures

**Figure 2:**
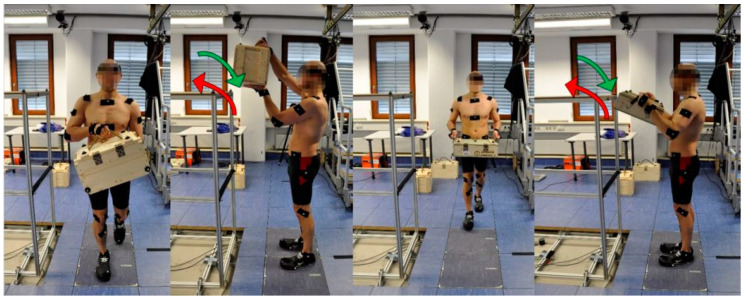
Subject performing motion sequences T1–T6 in the motion laboratory. From left to right: carrying box A (T1), lifting (T2, red arrow) and lowering (T3, green arrow) box A, carrying box B (T4), lifting (T5, red arrow) and lowering (T6, green arrow) box B.

Six representative motion sequences that occur during a lifting task were selected, recreated in the motion laboratory, and carried out by the subjects. The sequences comprised the carrying (T1), lifting (T2), and lowering (T3) of box A with one hand holding a top handle and the other hand stabilizing the box from below, as well as the carrying (T4), lifting (T5) and lowering (T6) of box B with both hands on the lateral handles of the box (see Figure 2). All of the six resultant motion sequences were carried out with overall box weights of 21 kg and repeated up to three times to ensure a reliable database for analysis. The test subjects were encouraged to train the working motions in advance based on videos of actual military logistics employees working in a real environment.

### 2.4. Biomechanical Modeling of the Motion Sequences

Each motion sequence was isolated based on a characteristic kinematic pattern of optical markers. For the carrying scenarios, a complete walking cycle was extracted by detecting the initial and terminal stance phase (heel and toe markers on the same height). For the lifting and lowering sequences, the vertical velocity of the markers was used to identify the points in time when the box reached its highest and lowest positions. These were then used to segment the lifting and lowering motion sequences. The motion sequences for each subject were initially preselected in QTM (Qualisys Track Manager, 2019.2, Qualisys, Gothenburg, Sweden) and subsequently processed in the AnyBody Modeling System^TM^ (AMS, AnyBody Technology A/S, Aalborg, Denmark) to obtain a holistic comprehension of the human kinetics within the considered manual handling scenarios. In the AMS, a multibody model, consisting of a human body model (joints, bone segments and muscles) and possibly other objects, is driven by measured or modeled motion data [50]. The algorithm then computes inner human body JFs and MAs based on the motion and load data by solving an optimization function, which aims to minimize overall muscle activities within an inverse dynamics approach [50].

In the AMS, the human model of the AMMR (AnyBody Managed Model Repository) Version 2.2 combined with the shoulder model of [51] was used and connected with the boxes via the hands. The CAD models of the boxes were modeled in SolidWorks (SolidWorks 2018, Dassault Systèmes, Vélizy-Villacoublay, France) and subsequently translated via Exp4SOLIDWORKS into the AMS. Marker positions on the test subjects and the boxes were used to solve each model’s kinematics. The mechanical interface between the hands and the boxes was modeled by six infinitely strong bidirectional rotational and translational contact functions (*AnyReacForce*, AMS) to share the load of the box symmetrically between both hands. The ground reaction forces at the feet of the human model were quantified by predictive ground reaction force elements. Those comprise points on the feet and a ground plane zone. Once target points are located inside and moved relative to the ground plane, normal and friction forces arise which counteract the model’s resulting forces and are considered in the aforementioned optimization function. For more information about the validated force prediction method, see [52].

The external weight (21 kg) was assigned separately to the modeled box, and based on the kinematics, a kinetic solution was simulated for each dataset.

### 2.5. Motion Sequence Evaluation and Selection

The six motion sequences in the lifting process were analyzed to determine which of the activities led to the highest MAs and JFs and, therefore, was a priority for support. This activity was subsequently used in further simulations to compare different exoskeleton concepts in terms of their biomechanical effectiveness. 

For the evaluation, biomechanical stress parameters were chosen that allowed for a holistic analysis. These included measures for peak and cumulative stress in selected body segments. The body areas were chosen based on subject feedback during the trials (loaded areas: arms, shoulders, wrist, lower back and neck) and complemented by areas that are frequently affected by musculoskeletal diseases (e.g., lower back, hip and knee joints) to obtain a more holistic impression of the overall stresses in the body. The envelopes of MA of muscle groups (including muscles of the left and right side of the body) and their integral over time, served as a measure of cumulative muscle stress. The considered muscle groups included major muscles in the arms (M. Biceps Brachii, M. Triceps Brachii, M. Brachialis, M. Brachioradialis), the shoulders (M. Deltoideus, M. Supraspinatus, M. Infraspinatus, M. Teres Major, M. Teres Minor, M. Subscapularis, M. Coracobrachialis), the neck (M. Longus Colli, M. Longus Capitis, M. Scalenus, M. Splenius, M. Semispinales Capitis, M. Semispinales Cervicis, M. Longissimus Capitis, M. Longissimus Cervicis, M. Multidus Cervicis), the lower back (M. Multidi, M. Erector Spinae, M. Quadratus Lumborum, M. Semispinales, M. Spinalis), the abdomen (M. Transversus, M. Rectus Abdominis, M. Obliquus Externus, M. Obliquus Internus), the lower extremities (all leg muscles included in the AMMR Body model) and an envelope over muscles of the whole body. In addition, the maximum JFs (vector magnitudes of resultant left and right JFs and the magnitude of compression force between the lumbar vertebrae L4 and L5) of the selected joints (knee joint, hip joint, L4/L5, glenohumeral joint, elbow joint, wrist) were derived. Mean values and standard deviations for the biomechanical stress criteria (MA integrals and maximum JF of the aforementioned muscle groups and joints) were calculated over the three subjects.

To identify the most strenuous motion sequence, points were given to the motion sequences for each biomechanical stress criterion based on their relative ranking (from one for the motion sequence with the lowest value up to six for the one with the highest value), using mean values of the biomechanical stress criteria. The points for all criteria were added up for each motion sequence to generate an overall score. The motion sequence with the highest score was interpreted as the most strenuous one and subsequently used for the investigations with the different exoskeleton concept variants.

### 2.6. Exoskeleton Concepts

**Figure 3:**
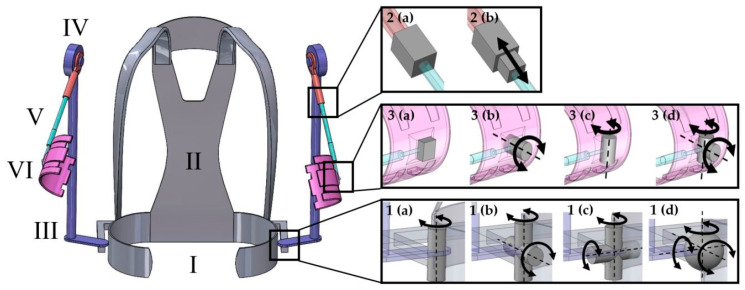
Model of the exoskeleton, including the hip belt (I, grey) and back plate (II, grey), the load-bearing structures (III, violet) between the hip belt and torque-generating joint (IV), the upper arm bracing (VI, pink), as well as the proximal (red) and distal (cyan) parts of a structure (V) connecting the torque-generating joint and arm bracing. Black squares show the three possible joint positions per side. In the respective magnifications (black rectangles) the joint variants are illustrated: The hip connection (between grey and violet structures) can include a revolute joint (1 (a)), universal joints (1 (b), first axis: vertical axis or 1 (c), first axis: vertical axis)) or a spherical joint (1 (d)). The parts (red and cyan) of the structure between the torque-generating joint and arm bracing can be connected rigidly (2 (a)) or via a prismatic joint (2 (b)). The connection between the distal part of the structure and the arm bracing can be fixed (3 (a)), include two types of revolute joint (3 (b) and (c)) or a universal joint (3 (d), first axis: vertical axis). Grey blocks, cylinders, and spheres symbolize the respective joints. Additional axes (dotted lines) and black arrows indicate the possible axes of translation (2 (b)) or rotation.

The preselected exoskeleton concept for heavy lifting support is presented in Figure 3. It comprises a hip and back connection (Figure 3, I and II) to which two joints are attached, one to the left and one to the right side (Figure 3, 1(a)). Both joints contained one rotational degree of freedom (DOF) parallel to the human longitudinal axis (1(a)), and both merged into a load-bearing structure (III), which led laterally to a torque-generating joint (IV) near the shoulder. From there, another load-bearing structure (V) led through a prismatic joint 2(b) and an additional rotational joint 3(b) to an upper arm bracing (Figure 3, VI)). The torque-generating joint was designed with passive torque generation (using springs), as can be found in so-called passive or semi-active exoskeletons [36].

The next step was optimizing the concept’s basic architecture with regard to its biomechanical effectiveness. For this purpose, three locations for possible joint variations were defined on each side: One on the hip belt, one at the upper arm bracing, and one between the torque-generating joint and the upper arm bracing. The joints were placed at the exact same location on the exoskeleton in every configuration. A selection of suitable joints was made for all three joint positions based on known exoskeletons and other potentially useful combinations. The possible joints are illustrated in Figure 3. In this way, four variants each for arm and hip connection and two variants for the connection between arm bracing and torque-generating joint were defined, resulting in a total of 32 exoskeleton combinations. Variants that could not be simulated (for example, because the combined human-exoskeleton model was kinematically over-constrained) were excluded from further analysis. All others were examined concerning the parameters presented in Section 2.8.

### 2.7. Modeling of the Exoskeleton-Human System and Interaction

One exemplary dataset (height: 1.76 m, weight: 70.4 kg) of one subject model in combination with the most strenuous lifting task was selected for further analysis, and the aforementioned exoskeleton concepts were included in the AMS and virtually interfaced with the human model (AMMR Version 2.4.2, including the shoulder model of [51]). The exoskeleton was modeled using SolidWorks and exported using the Exp4SOLIDWORKS tool. The exoskeleton structures, including the hip, back, and arm connections, were modeled as rigid bodies in CAD (total weight: 5.1 kg). Joints in between them were not designed in SolidWorks. However, coordinate systems were defined in the segments with centers positioned at the destined joint locations. Two coordinate systems each were then used in the AMS to define the respective joint with varying DOF, as illustrated in Figure 3. All joints were modeled as ideal joints, which are arbitrarily small (no requirements for mechanically sufficient strength) and not affected by friction. The rotational axes of universal and spherical joints all intersect in one center of rotation. In addition, DOF were not restricted in their range of motion.

**Figure 4:**
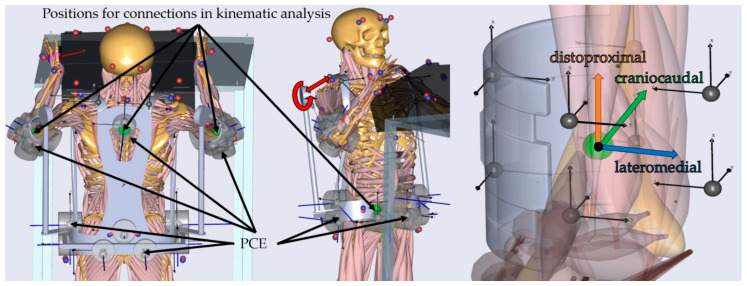
(**Left**): Two different angles from the exoskeleton connected to the human model in the AnyBody Modeling System^TM^ (AMS). The exoskeleton (grey) is connected with the human body model with *AnyKinExtraDrivers* (positions pictured with green spheres) in the kinematic analysis and with predictive contact elements (PCE, grey transparent cylinders) in the inverse dynamic analysis. They are positioned at the arms (16 elements), hip (10 elements), and back (one element). Arrows show exemplary positions of PCE at the sides, front, and back of the hip belt, the back, and the arm bracings. Blue lines show normal and friction forces currently acting on the PCE. Torques generated by the torque-generating joints and spring forces between the back plate and torque-generating joints are depicted with red arrows. (**Right**): Arm and Arm bracing (transparent) with coordinate system defined in its center with three axes: the distoproximal axis (along the arm longitudinal axis), the craniocaudal axis (points to the bottom when shoulder flexion is 90°) and the lateromedial axis (points to the sagittal plane when shoulder abduction is zero). The position of the *AnyKinExtraDrivers* is in the center of the coordinate system (green sphere). Positions of the PCE are illustrated by eight black coordinate systems around the arm bracing.

In order to solve the kinematics in the AMS, marker driver functions (*AnyKinMarkerDriver*) were implemented for the models of the human body and the box based on the imported optical motion capture data. To solve the kinematics of the exoskeleton, kinematic drivers (*AnyKinExtraDriver*) were defined in the connection between the human and the exoskeleton model (in the arm, hip and back interfaces) in order to constrain the motion between the human and the exoskeleton model (Figure 4). Constraints were set as *hard* and *soft* as needed, defining a DOF restriction to be fixed strictly (*hard*) or to be considered with less priority by the kinematics solver if needed (*soft*). All six DOF at the hip and the back connection, respectively, were set to *hard*. For the arm connection, the three translational DOF were set to *hard*, and the three rotational DOF were set to *soft*.

To kinetically interface the exoskeleton and the human body model during the inverse dynamic analysis, predictive contact elements (PCE) were utilized for the arm, hip, and back interfaces. PCE work similar to the aforementioned ground reaction forces (see Section 2.4, also [52]) and comprise a target point on one object and cylindrically shaped contact zones on another object. When the target point is located inside and moved relative to the cylindrical zone, normal and friction forces arise and are considered in the muscle recruitment [52]. The implemented class allows for adjusting the static friction coefficient, the normal and friction directions, and the maximum strength of the normal force component. The hip belt was connected using 10 PCE positioned at the belt segment of the exoskeleton with corresponding points on the pelvis segment of the human model (Figure 4). Another PCE was positioned on the back segment (same rigid exoskeleton segment) with a corresponding point on the thorax to counteract external torques. Finally, eight PCE were defined in each arm bracing (Figure 4). They were positioned and oriented so that forces perpendicular to the humerus and torques around those axes could be transferred via forces in the normal direction of the PCE. Forces parallel to the arm’s longitudinal axis and torques around it were transferred via the friction component of the PCE. The maximum strength was set to 10.000 N, and the friction coefficient to 1.0 for all PCE. 

The exoskeleton support modeling took place in two steps. In the first step, the base concept was utilized, and the support was modeled using artificial kinetic functions (*AnyReacForce*) in the torque-generating joint, creating a torque between adjacent segments (Figure 4). In this manner, the torque-generating joint acted as an infinitely strong actuator, and the optimization algorithm utilized it to minimize muscular efforts across all muscles. The torque generated over the angle of the torque-generating joint was defined then as the ideal target torque curve. In active exoskeletons, motors could potentially constitute the ideal non-monotonic torque curve. However, passive shoulder exoskeletons show mostly monotonic torque curves that can be described by a polynomial of degree two (e.g., [53,54,55,56]). To allow for an easier mechanical implementation in the future, an approximation of the target torque curve in the form of a polynomial of degree two ($y=27.6381\;\mathrm{Nm}+0.5570\;\frac{\mathrm{Nm}}{{}^{\circ}}*x-0.0036\;\frac{Nm}{{\left({}^{\circ}\right)}^{2}}*x^{2}$; x: angle in °, y: torque in Nm) was conducted and used for all simulations in order to ensure good comparability between concepts.

In addition, two relatively small spring forces (spring constant: 10 N/m) were introduced in the inverse dynamic analysis. These acted between points on the back segment and the torque-generating joint, pulling both together (Figure 4). One can think of them as elastic rubber bands between the back segment and the torque-generating joint that would pull exoskeleton structures closer to the human body in reality.

### 2.8. Exoskeleton Concepts Analysis

For the comparison between the exoskeleton concept variants, forces and MAs in the back and shoulder were used as the main criteria since the exoskeleton is intended to relieve these body parts in particular. MAs included envelopes of the corresponding muscle groups (including muscles from the left and right side, compare Section 2.5), JF included the average forces between the left and right side. For better quantitative comparison, mean values were calculated over the motion sequence.

Other relevant body areas were investigated for additional stresses but were not included in the final selection process of the most promising concepts. Instead, further parameters, such as forces and moments acting in the upper arm interfaces, were considered because of their potential influence on the comfort and functionality of the exoskeleton. Forces pushing perpendicular to the arm’s longitudinal axis from behind or below (forces in the craniocaudal direction, see Figure 4), as well as torques creating forces in the same or opposite direction (torques around the lateromedial axis, see Figure 4) help in lifting the arm and are labeled supporting forces and torques hereinafter. In contrast, other forces and torques that do not help the worker are denoted as parasitic forces and torques. Forces along and torques around the arm’s longitudinal axis (hereinafter denoted as the distoproximal axis, see Figure 4) lead to shear forces in the skin. Forces in the lateromedial axis and torques around the craniocaudal axis push the arm inward or outward. 

To determine these force and torque parts, normal and friction force components of the PCE were summed in the respective directions. Torques around the axes were calculated from the normal acting and frictional forces and the respective lever arms to those forces. For parasitic forces (along the distoproximal or lateromedial axis) and torques (around the distoproximal or craniocaudal axis), absolute values were averaged over the motion sequence and both arm interfaces. For supporting torques (around the lateromedial axis) and forces (along the craniocaudal axis), we were not only interested in the magnitude but also if they have parts in different directions (positive and negative). Hence, originally signed values were used. However, values were mirrored to appear mostly in the positive direction for better readability of the graphs.

Finally, a ranking process was conducted following the same procedure described in Section 2.5. For this purpose, points ranging from one to six (higher values receive more points) were allocated to each concept in every stress criterion and added up for each concept to generate an overall score. The concepts with the lowest overall score were interpreted as the concepts with the highest biomechanical relief potential (or biomechanical effectiveness).

## 3. Results

### 3.1. Motion Sequence Evaluation and Selection

Table 1 summarizes the results for the biomechanical stress criteria (MA time integrals and maximum JFs) for each motion sequence (T1–T6). Based on the obtained results, an overall score was computed for each motion sequence using the procedure described in Section 2.5.

The MAs showed mean values between 54%s and 90%s over the whole body envelope. The lower extremities showed mean values between 11%s and 28%s. In the abdomen values between 21%s and 39%s were determined, which were similar to the mean values in the back (21%s–37%s). Mean MAs in the neck were rather low (11%s–24%s) in contrast to the shoulders (23%s–62%s) and the arms (50%s–87%s). Averaged maximum joint forces in the knee ranged from 2.8 kN to 4.3 kN and were similarly high in the hip joint (3.0 kN–4.3 kN). The lumbar segment L4/L5 showed average maximum forces between 2.0 kN and 3.2 kN. In the glenohumeral joint, values between 1.8 kN and 3.7 kN were observed, whereas the wrist (0.7 kN–1.7 kN) and elbow (0.9 kN–1.4 kN) showed considerably lower values.

The biomechanical stress criteria show that most body regions were most stressed (see maximum values of each row) during the two lifting activities (T2 and T5). The results of the overall rating underline these findings. The lifting activity lifting box B (T5) was identified as the most stressful activity, followed by lifting box A (T2) and the two carrying activities (T1 and T4), with the lowering sequences (T3 and T6) as the least stressful activities. For the subsequent comparison of different exoskeleton concepts, lifting box B (T5) was selected as the representative motion sequence for the maximum strenuous load case (compare Figure 2).

The lifting of heavy objects up to shoulder level led to large muscle loads in shoulders (56 ± 15%s), the arms (87 ± 7%s, especially upper arm flexors) and subordinately also in the back (35 ± 3%s),abdomen (36 ± 3%s), neck (24 ± 12%s) and lower extremities (22 ± 3%s). Maximum resultant JFs in the joints reached 4.3 ± 1.6 kN in the knee, 4.2 ± 0.2 kN hip and 3.2 ± 0.7 kN between L4 and L5. The glenohumeral joint showed forces of 3.7 ± 1.8 kN, the elbow of 1.2 ± 0.4 kN, and the wrist of 1.7 ± 1.2 kN.

### 3.2. Exoskeleton Concepts Analysis

Six of the 32 exoskeleton variants could be simulated under the given conditions and underwent kinematic and inverse dynamic analysis with plausible results. All variants in which the beam between the arm bracing and torque-generating joint had a fixed connection (Figure 3, 2(a)) could not be solved kinematically. Likewise, the kinematic analysis of all variants in which the arm connection was fixed (Figure 3, 3(a)) could not be solved either. Of the remaining 12 variants, in another three, analysis was aborted, and one showed biomechanically unfavorable results, leading to its exclusion. Two variants showed plausible results over the first 80% of the motion sequence. However, short-term high peaks occurred in some biomechanical stress parameters, which would have distorted mean values, which is why they were excluded from further analysis.

The results of the remaining six variants are analyzed below. Henceforth, they are denoted by their joint configuration (e.g., H: R.cc–A: R.lm), utilizing joint names from Table 2. Numbers are given as mean values over the motion sequence and the left and right sides of the body. MAs show the percentage of the maximum possible MA of the muscle.

**Figure 5:**
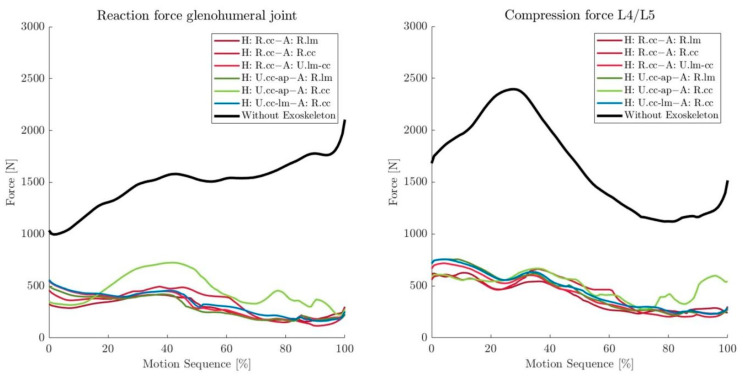
(**Left**): Resultant forces in the glenohumeral joints (averaged between left and right of one subject) over the motion sequence T5 without (black) and with exoskeleton concept variants (colored). (**Right**): Compression forces between L4 and L5 over the motion sequence T5 without (black) and with exoskeleton concept variants (colored).

All variants reduced the resultant forces in the glenohumeral joint over the course of the motion sequence from an average of 1495 N without the exoskeleton to averages between 287 N and 461 N (see Figure 5 left and Table 3). The variants with a revolute joint in the hip connection (H: R.cc–A: R.lm, H: R.cc–A: R.cc, H: R.cc–A: U.lm-cc), as well as configurations H: U.cc-ap–A: R.lm and H: U.cc-lm–A: R.cc showed considerably lower average JFs (287 N–334 N) than the configuration H: U.cc-ap–A: R.cc (461 N, see Figure 5 left, Table 3). Similar tendencies were observed for the shoulder MAs. Mean activities were reduced from 37% of the maximum MA without the exoskeleton to 13–15% of the maximum MA in variants H: U.cc-ap–A: R.lm, H: U.cc-lm–A: R.cc and all variants with a revolute joint in the hip connection (H: R.cc, see Table 3). Variant H: U.cc-ap–A: R.cc showed a mean MA (27%) that was distinctly lower than the configuration without the exoskeleton but higher than the other variants.

Back MAs and L4/L5 compression forces showed similar trends as the shoulder, with the same variant (H: U.cc-ap–A: R.cc, JF: 502 N, MA: 10%) showing slightly higher values than the rest (MA: 9–10%, JF: 402–461 N) (see Figure 5 right, Table 3). Nevertheless, they were strongly reduced compared with loadings without exoskeleton support (MA: 28%, JF: 1665 N).

In other parts of the body, concept-related differences were smaller, which is why we only show exemplary data averaged over all concepts (using previously calculated mean values determined over the left and right side and the course of the motion), including their standard deviations as a measure of dispersion. The mean abdominal MAs were reduced with an exoskeleton (mean MA: 14%, std.: 2%) compared with the simulation without an exoskeleton (27%). However, activities in the arm flexors and lower extremities slightly increased with the exoskeleton concepts. MAs in the lower extremities rose from an average of 18% without an exoskeleton to 24% (std.: 0.5%) with an exoskeleton. Activities of the arm flexors rose from 58% to 64% (std.: 2%). In both areas, this unfavorable behavior occurred, especially during small shoulder flexion angles in the first half of the motion sequence.

**Figure 6:**
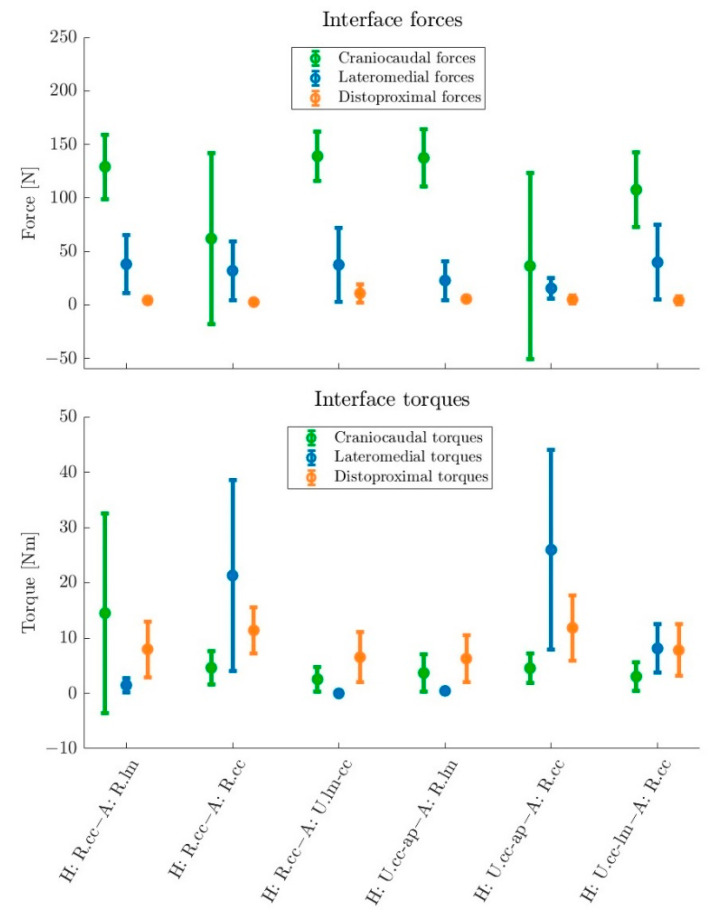
Mean forces and torques in the arm interface with standard deviations for six concepts averaged over left and right arm bracing and the motion sequence T5. The diagrams show absolute torques around the craniocaudal (green) and distoproximal (orange) axis and absolute forces in the direction of the lateromedial (blue) and distoproximal (orange) axis. For craniocaudal forces and lateromedial torques averages of signed values are shown.

Besides MA and JF, reaction forces and torques in the human–exoskeleton arm interfaces were investigated. In Figure 6, the mean value (averaged over the motion sequence) and corresponding standard deviation of the force and torque for each of the three axes in the arm bracing are given. These include the main supporting forces (craniocaudal) and torques (lateromedial) as well as parasitic forces (distoproximal, lateromedial) and torques (distoproximal, craniocaudal).

The variants H: R.cc–A: R.lm (129.1 ± 30.0 N), H: R.cc–A: U.lm-cc (138.9 ± 23.0 N), H: U.cc-ap–A: R.lm (137.4 ± 26.7 N) and H: U.cc-lm–A: R.cc (107.6 ± 34.9 N) showed high mean supporting craniocaudal forces with relatively small deviations (Figure 6). The variants H: R.cc–A: R.cc (61.9 ± 80.0 N) and H: U.cc-ap–A: R.cc (36.4 ± 87.2 N) showed more dispersed results, incorporating greater areas of negative values and subsequently showing smaller mean values. However, supporting lateromedial torques (H: R.cc–A: R.cc (21.3 ± 17.2 Nm), H: U.cc-ap–A: R.cc (25.9 ± 18.0 Nm)) were much higher in these variants (see Figure 6, Table 3). H: U.cc-lm–A: R.cc (8.1 ± 4.4 Nm) also shows slightly higher torques compared with the remaining three concepts, which had almost zero lateromedial torque (H: R.cc–A: R.lm (1.4 ± 1.3 Nm), H: R.cc–A: U.lm-cc (0.0 ± 0.1 Nm,), H: U.cc-ap–A: R.lm (0.4 ± 0.3 N)).

Lateromedial parasitic forces that push the arm outward or inward were smaller than the craniocaudal forces (see Figure 6, Table 3). The variants H: R.cc–A: R.lm (38.1 ± 27.3 N), H: R.cc–A: R.cc (31.9 ± 27.3 N), H: R.cc–A: U.lm-cc (37.5 ± 34.7 N) and H: U.cc-lm–A: R.cc (39.7 ± 35.0 N) show similar high lateromedial forces. Slightly smaller forces were found in H: U.cc-ap–A: R.lm (22.7 ± 18.3 N) and H: U.cc-ap–A: R.cc (15.4 ± 9.7 N). Torques around the craniocaudal axis result in forces that push one side of the arm bracing outward and the other inward. However, they were rather small over almost all concepts (H: R.cc–A: R.cc (4.6 ± 3.1 Nm), H: R.cc–A: U.lm-cc (2.5 ± 2.2 Nm), H: U.cc-ap–A: R.lm (3.7 ± 3.3 Nm), H: U.cc-ap–A: R.cc (4.5 ± 2.7 Nm)) with small standard deviations. In contrast, H: R.cc–A: R.lm (14.5 ± 18.0 Nm) showed rather high values and a high standard deviation due to large oscillations over the motion sequence.

Parasitic distoproximal shear forces along the arm longitudinal axis were relatively small in all concepts (H: R.cc–A: R.lm (4.2 ± 2.7 N), H: R.cc–A: R.cc (2.6 ± 2.1 N), H: U.cc-ap–A: R.lm (5.5 ± 2.3 N), H: U.cc-ap–A: R.cc (5.0 ± 3.7 N), H: U.cc-lm–A: R.cc (4.2 ± 3.6 N)), except for concept H: R.cc–A: U.lm-cc (10.8 ± 8.3 N) with slightly higher values and standard deviations (see Figure 6, Table 3). On the other hand, torques around this axis were notably substantial and differed between concepts. The two highest values were found in H: R.cc–A: R.cc (11.4 ± 4.2 Nm) and H: U.cc-ap–A: R.cc (11.8 ± 5.9 Nm), followed by the concepts H: R.cc–A: R.lm (8.0 ± 5.0 Nm) and H: U.cc-lm–A: R.cc (7.8 ± 4.7 Nm). The variants H: R.cc-A: U.lm-cc (6.5 ± 4.3 Nm) and H: U.cc-ap–A: R.lm (6.3 ± 4.3 Nm) showed the two lowest values (see Figure 6, Table 3).

In an overall comparison between concepts (see Table 3), considering the selected MAs, JFs, and parasitic forces and torques, the concept H: R.cc–A: U.lm-cc (22) achieved the best score, followed by H: U.cc-ap–A: R.lm (24), H: R.cc–A: R.lm (25) and subsequently H: R.cc–A: R.cc (28). Variant H: U.cc-lm–A: R.cc was rated with 30 points, and H: U.cc-ap–A: R.cc (39) was rated the worst.

## 4. Discussion

### 4.1. Motion Sequence Evaluation and Selection

The two lifting activities (T2 and T5) showed higher biomechanical stress criteria values and overall scores than the lowering and carrying motion sequences. T5 (see Figure 2) showed the highest values and was consequently selected as the most strenuous motion activity for subsequent exoskeleton concept simulations. One could argue that some criteria are more important than others and should, therefore, influence the calculation with a higher weighting. However, we do not expect the final outcome to change significantly since the differences between the different motion sequences are high. Nevertheless, with other weighting or other biomechanical stress criteria, T2 could be rated more strenuous than T5, which could also have an influence on the subsequent concept comparison.

In order to create an exoskeleton design concept with high biomechanical effectiveness for such motions, it is important to understand which areas of the body are heavily stressed and should, therefore, be relieved. In this case, a combination of peak forces in the joints and cumulative stress in the muscles was investigated in all body regions.

Our simulations showed peak JFs of 4.3 ± 1.6 kN in the knee and 4.2 ± 0.2 kN in the hip, which are considerably higher than forces in everyday activities such as walking (knee: 1.9 kN, hip: 2.0 kN, forces calculated from the mean subject weight of 74 kg and body weight factors 2.6 [57] and 2.7 [58], respectively). However, the cumulative MA was fairly low (22 ± 3%s) in the lower extremities, so we believe that an exoskeleton leg and hip support is beneficial but not the first priority for such a lifting task.

For the lower back, there are safety limits available (guideline values), the so-called “Revised Dortmund Recommendations” [59]. The average maximum compression force in the L4/L5 segment was 3.2 ± 0.7 kN in our simulations. This loading is not recommended for men over 50 or women over 40 years of age (guideline boundary value: 3.1 kN), which illustrates relatively high stress on the lower back [59]. MAs in the back (35 ± 3%s) and abdomen (36 ± 6%s) were also relatively high. Since the back is loaded in many other daily activities and is the most common cause for work-related musculoskeletal disease complaints in the EU [1], it is advised to also support this part of the body.

In the glenohumeral joint, we found peak forces of up to 3.7 ± 1.8 kN, compared with resultant forces of up to 1.1 kN (body weight factor: 1.5 [60]) when lifting a 1.5 kg weight in front of the body (≥90° shoulder flexion) with the arm stretched out. In our simulations, the cumulative shoulder MAs also showed high values (56 ± 15%s). In combination, we conclude that a shoulder support is essential for supporting heavy lifting from hip to shoulder level.

For the elbow and wrist, we did not find any convincing comparative values from in vivo measurements in the literature. Nevertheless, high MA in the arms (87 ± 7%s, especially upper arm flexors) indicate the importance of also supporting the arms during heavy lifting. As a result, we recommend relieving, in particular, the arms and shoulders (as we previously stated [47]) and, secondarily, also the back during heavy lifting activities up to shoulder height.

However, to create an exoskeleton with good user acceptance, not only is a biomechanically sound concept required, but the system also needs to meet demands regarding comfort and usability. In order to reduce the system’s complexity and potential weight, no support for the arms (wrist, forearm, elbow, upper arm) was included in the first development stage of the exoskeleton, and it, therefore, supports only the shoulders and back down to the hips. Nevertheless, we recommend extensions for added arm support in the future since high cumulative muscle stresses were determined in the arms.

### 4.2. Exoskeleton Concepts Analysis

23 of the 32 variants could not be simulated completely. Investigation of the corresponding joint configurations led to the following findings. We found that the concepts require a prismatic joint (linear DOF) between the actuator and the upper arm bracing (Figure 3, 2(b)) in order to provide kinematic compatibility with the performed motion and complete kinematic analysis. Furthermore, no variants that did not have at least one rotational DOF in the connection to the arm bracing could be simulated. In reality, these concepts may still work because the connection between humans and the exoskeleton is more flexible than in our simulations. However, we expect high interaction forces, possibly causing discomfort or slippage. Another three variants were simulated but yielded either unfavorable biomechanical results (in one variant) or showed implausible high peaks over a short duration of the motion sequence (in two variants). The latter were excluded because the peaks would influence mean values over the motion sequence, thereby falsifying the outcome. The two concepts had a particularly large number of DOF, but one or more were redundant during parts of the motion sequence. We believe that the additional DOF allowed for motions at the beginning of the sequence, leading to model orientations in which further motions were kinematically restricted. In the kinematic analysis, some *soft* constraints in the human exoskeleton connection (see Section 2.7) were ignored to enable the motion. However, in the inverse dynamic analysis, the motion of the human body model is still restricted by the motion of the exoskeleton structures, which are connected through the PCE. Those create forces to the upper arm, which work against the motion of the human model assigned in the kinematic analysis. Body muscle recruitment is increased until an equilibrium is reached in the optimization algorithm, which incorporates the body muscles and the PCE [52]. The consequences are constraining forces in the PCE and joints through additional muscle activation. This happens to a smaller extent whenever human body motion is constrained and is the reason for constraining forces in the exoskeleton body interfaces (compare Figure 6). On the other hand, this allows for the movement of models that are kinematically overconstrained during parts of the motion sequence. In the two variants, this effect built up particularly high. In reality, there would probably also be increased constraint forces in these positions. However, they would cause deformation of the soft interfacing body tissues and consequently not build up such high peaks as we observed in the simulation. The result would probably be increased discomfort, but muscular relief should still be present to a higher degree.

The remaining six investigated concepts could strongly support the body during heavy lifting activities. All concepts reduced mean JFs by at least 69% in the glenohumeral joint and 70% between L4 and L5. MAs in the lower back were reduced by at least 63%. In the shoulder muscles, reductions of at least 59% could be achieved when excluding the concept with the worst overall score (H: U.cc-ap–A: R.cc). Although these results give a first indication of the quality of musculoskeletal relief, one must consider absolute values carefully since they depend on many factors. This includes, amongst others, anthropometric data of the human model, dimensions of the exoskeleton structures, muscle strength values and muscle recruitment strategies, and the connection between the human model and the exoskeleton. For example, the arm interface’s position and strength values of and distances between different PCE in the said interface would influence reaction forces and torques. Additionally, the hip and back connections influence which fractions of the load are transferred via exoskeleton or human structures and thereby alter the musculoskeletal relief of the back considerably. Nevertheless, all parameters were chosen carefully in order to generate plausible results.

On the other hand, our results also show that the concepts come with some disadvantages. For example, the lower extremities were subjected to approximately 25% more load than without the exoskeleton. However, it should be noted that here the MAs were generally relatively low, so the additional load is probably less problematic. The situation is different for the arm muscles. Here, an average increase of 9% was observed in MA. This was comparatively small compared with the large reductions in shoulder and back stresses, so there was—all in all—a positive tradeoff. However, the activity of the arms was already high, and any further increase should be avoided. One possibility would be to reduce the support torque but at the expense of shoulder and back relief. In the long term, however, one should implement an exoskeleton extension beyond the elbow to ensure elbow and upper arms relief during heavy lifting tasks. It should be noted that the additional load could originate in part from parasitic forces in the human–exoskeleton interfaces, pushing and bending the arms in various directions, which must be counteracted by the muscles. Due to tissue softness, this effect could be smaller in reality.

**Figure 7:**
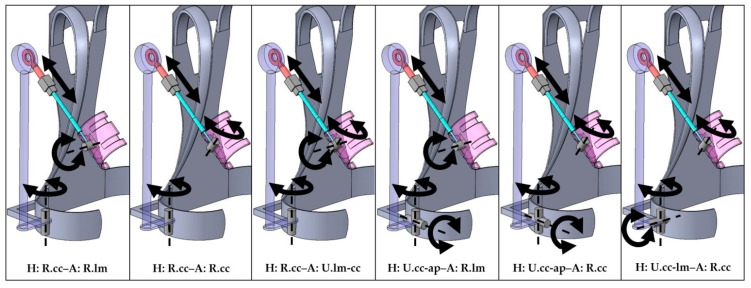
The six final exoskeleton variants are depicted with their respective joints (symbolized by grey blocks and cylinders). Corresponding DOF (axes of translation or rotation) are depicted with black arrows and dotted lines.

When looking at the MAs and JFs of the individual concepts, it is noticeable that all concepts with a revolute joint in the hip connection (Figure 7, H: R.cc–A: R.lm, H: R.cc–A: R.cc, H: R.cc–A: U.lm-cc) performed well, with relatively low JFs and MAs. Good results were also generated in the concept with a universal joint that allowed for additional outward/inward tilting in the hip connection, combined with a joint for forward/backward tilting in the arm connection (H: U.cc-ap–A: R.lm). This was also true for the concept that allowed forward/backward tilting in the hip connection, and inward/outward rotation in the connection to the arm interface (H: U.cc-lm–A: R.cc). In both cases, all DOF were oriented perpendicular to each other during most of the motion.

The support force was high in most variants. However, in variants with no rotational DOF allowing for forward tilting of the arm bracing (all variants with a revolute joint with a craniocaudal axis in the connection to the arm bracing), larger torques were transmitted around the lateromedial axis. This can be observed particularly in H: R.cc–A: R.cc and H: U.cc-ap–A: R.cc (see Figure 7) in which strongly fluctuating low average forces occurred in the craniocaudal direction, while high lateromedial torques emerged. To a smaller extent, this phenomenon can also be seen in variant H: U.cc-lm–A: R.cc. Here, however, the DOF in the hip interface that allowed forward tilting of the load-bearing structures of the exoskeleton relativized this effect. Such a torque around the lateromedial axis can reduce the torque in the shoulder and consequently occurring JFs and related MAs. However, compared with a pure force perpendicular to the arm connection, moments cause undesirable edge pressures in the arm connection, which could lead to discomfort [61]. The magnitude of these torques may be smaller in reality since tissue deformations reduce occurring moments. Nevertheless, concepts that generate increased moments in the interfaces should be avoided. Concepts H: R.cc–A: R.lm, H: R.cc–A: U.lm-cc, and H: U.cc-ap–A: R.lm (see Figure 7) all explicitly allowed a degree of rotational freedom in the connection to the arm interface, which resulted in minimal lateromedial moments in the arm bracing.

If we look at the variables we defined as parasitic torques and forces in the arm connection, the following is noticeable. Forces acting along the longitudinal axis of the arm were almost zero. This is highly desirable, as such forces would result in undesirable motion or uncomfortable shear forces along the skin [62]. The low forces are plausible and justified by the prismatic joint between the arm connection and the torque-generating joint, which is almost parallel to the longitudinal direction of the arm. However, it is noticeable that the moments around the distoproximal axis were relatively high across all concepts. This was due to the boundary conditions in our simulations that did not enable a rotational DOF around the arm longitudinal axis in the arm interface. In reality, some rotation around this axis is possible due to slippage and tissue deformation. Therefore, such high torques would probably not occur. We did not include this DOF since it does not exist unrestricted in reality. Twisting the arm is possible, but not arbitrarily far, and resistance would still occur. However, future simulations could include this DOF with an additionally implemented realistic rotational stiffness during inverse dynamic analysis.

Lateromedial forces that push the arm outward or inward were generally rather high over most variants and varied more between concepts. In particular, variants that did not allow the exoskeleton to tilt outward/inward at the hip interface connection confined the motion and thereby created constraining forces in the marker-driven model (Figure 7, H: R.cc–A: R.lm, H: R.cc–A: R.cc, H: R.cc–A: U.lm-cc, H: U.cc-lm–A: R.cc). Craniocaudal torques, originating from oppositional oriented lateromedial forces in different PCE, tended to be small, except for variant H: R.cc–A: R.lm. In this concept, the connection to the arm bracing did not include a joint that allowed for outward or inward tilting of the exoskeleton relative to the arm. In combination with the restricted outward/inward tilting in the hip connection, the possible motion of the human model is strongly confined, leading to high craniocaudal torques and lateromedial forces.

Finally, one can draw the following conclusions concerning the occurring parasitic forces and moments. There should be a linear DOF (prismatic joint) in the longitudinal direction of the arm between the arm connection and the torque-generating joint to minimize distoproximal constraint forces. In addition, there should be at least one further rotational joint in the connection to the arm interface. Particularly advantageous are those joints that allow the arm shell to tilt forward as this minimizes lateromedial constraining torques. In order to reduce lateromedial forces on the arm, a DOF in the hip interface is also useful as it allows the exoskeleton structures to tilt outward. The exoskeleton variant H: U.cc-ap–A: R.lm (see Figure 7) combines all these. However, slightly lower shoulder and back relief relativizes this advantage in the latter concept compared with others.

The results of the overall assessment reflect this as well. Concepts H: U.cc-ap–A: R.lm and H: R.cc–A: U.lm-cc showed the best valuations. The latter showed a slightly better overall rating only due to negligibly lower MAs. Comparing MAs and JFs of both variants, we conclude that both exhibited similar biomechanical effectiveness. However, H: R.cc–A: U.lm-cc showed considerably higher distoproximal and lateromedial constraint forces. Subsequently, H: U.cc-ap–A: R.lm proved to be the most advantageous combination to effectively support the investigated lifting activity while showing minimal constraint forces. Hence, we expect the concept to have great potential regarding its biomechanical effectiveness and comfort.

The proposed workflow provides a good opportunity to generate qualitative comparisons between exoskeleton concepts and allows for an initial assessment. However, there are limitations to a comprehensive concept optimization. Validation and verification of musculoskeletal models is a complex issue and should be considered an ongoing iterative process [63]. Parts of the Anybody human body model have been validated before regarding different aspects. For example the ability of the Anybody human body model to accurately determine vertebral loads was validated, comparing calculated and in vivo measured lumbar pressures [64]. However, our modeled combination of human model and concept variants has not yet been validated experimentally. Consequently, future research should focus on the model’s validation of the mechanical interface, e.g., based on the work of Chander et al. [65]. In addition, occurring interface moments could not be reduced through tissue deformations to an extent, as is the case in reality, and were, therefore, likely overestimated. Consequently, future models should attempt to implement realistic stiffness parameters for the human–machine interface (e.g., via force-dependent kinematics [66]) to better estimate more realistic acting forces and torques. Occurring load peaks could also be reduced in this way. It is also difficult to classify constraint forces in terms of the discomfort they cause since we found no relevant literature data, linking acting arm interface forces to discomfort sensation. Naturally, constraining forces and torques should be as low as possible; however, parasitic loads always exist in one way or another due to inevitable misalignments between human and machine joints [67,68].

Furthermore, new approaches should be investigated to allow for the stable kinematic simulation of concepts with under-constrained and especially more over-constrained joint combinations. Concepts that could not be successfully simulated here may well be advantageous in terms of their practicality of use. For example, fewer DOF can reduce the complexity, weight, and costs of an exoskeleton. A simulation with realistic interface stiffness and discomfort limits would allow for a better comparison of cheaper, lighter, but possibly slightly less comfortable exoskeletons with more sophisticated ones.

Finally, one should keep in mind that we investigated the early stages of exoskeleton designs concerning their biomechanical effectiveness and body interaction during a specific task. While this helps to identify biomechanically sound concepts, further research and development is, of course, necessary to understand if said concepts can also meet the end user’s needs concerning comfort, usability, range of motion, and others. However, these parameters are not influenced by the kinematic structure only, but also by other conceptual design decisions such as actuation (e.g., passive, semi-active or active) or details of engineered structures, interfaces, utilized materials and control strategies.

## 5. Conclusions

In this work, we identified particularly stressful motion sections during carrying and heavy lifting activities based on experimental motion capture recordings of realistic motion sequences and subsequent biomechanical analysis in multibody simulations. From our analysis, we conclude that especially the arms, shoulders, and back are highly loaded. Consequently, future exoskeletons should support these body areas.

A simulation-based workflow was used to evaluate and compare multiple simplified exoskeleton concept variants with respect to their biomechanical effectiveness and exoskeleton arm interface loads, which could inflict discomfort on the user. We found that the following DOF and joint combinations are most beneficial:

- A linear DOF (prismatic joint) in the arm longitudinal direction in front of the arm interface to reduce shear loads.
- A rotational joint in the connection to the arm interface allowing for forward/backward tilting of the arm bracing and minimizing lateromedial torques.
- A universal joint in the hip connection allowing for rotation parallel to the body axis and tilting outward/inward of the exoskeleton structures at the hip, thereby reducing lateromedial forces and improving the range of motion.
- A combination of these that allows for sound biomechanical unloading of the shoulders and back while parasitic forces and torques are effectively reduced.

Although the proposed workflow is already a promising tool for exoskeleton design analysis, it could be further improved by including a more elaborated model of the human–exoskeleton interface. For instance, more compliant connections between the human and exoskeleton model using realistic stiffness parameters derived experimentally could better take into account the effect of the soft tissues and yield a more accurate estimation of the interface forces and torques. Further experimental work is also needed to determine the limits of the loads that can be applied while ensuring comfortable interaction with the exoskeleton. Such findings could, in turn, be included in our simulation framework to provide quantitative thresholds for evaluating and comparing the concepts with respect to the possible occurrence of discomfort.

## Figures and Tables

**Table 1:** Mean values and standard deviations of selected muscle activities (MAs) integrals over time and maximum joint forces (JFs) for six motion sequences over three subjects (L4/L5 shows the magnitude of the compression force between lumbar vertebrae L4 and L5; the other forces are magnitudes of resultant JFs of left and right side). The last row shows the overall score for each of the motion sequences (higher values depict higher overall stress). Note that some previously reported values [47] are included in column T5 for better readability.

	Motion Sequence					
Biomechanical Stress Criteria	T1	T2	T3	T4	T5	T6
Muscle activities (MAs) [%s]						
Whole body	78 ± 6	82 ± 21	54 ± 16	72 ± 3	90 ± 13	45 ± 12
Lower extremities	28 ± 5	23 ± 8	15 ± 6	26 ± 4	22 ± 3	11 ± 1
Abdomen	27 ± 4	39 ± 10	30 ± 7	25 ± 1	36 ± 3	21 ± 4
Lower back	24 ± 3	37 ± 9	29 ± 9	22 ± 1	35 ± 3	21 ± 3
Neck	13 ± 3	19 ± 9	12 ± 2	17 ± 1	24 ± 12	11± 3
Shoulders	47 ± 16	62 ± 27	41 ± 17	27 ± 2	56 ± 15	23 ± 10
Arms	78 ± 7	72 ± 16	50 ± 14	72 ± 3	87 ± 7	44 ± 11
Joint forces (JFs) [kN]						
Knee	3.8 ± 0.5	3.7 ± 0.5	2.8 ± 0.8	3.4 ± 0.4	4.3 ± 1.6	2.8 ± 0.6
Hip	4 ± 1.2	3.1 ± 0.7	3 ± 0.5	4.3 ± 0.7	4.2 ± 0.2	3.3 ± 1.2
Glenohumeral	1.9 ± 0.5	2.7 ± 1.1	1.9 ± 0.2	1.4 ± 0.2	3.7 ± 1.8	1.8 ± 0.4
Elbow	1.2 ± 0.1	1.4 ± 0.4	1 ± 0.2	1.1 ± 0.1	1.2 ± 0.4	0.9 ± 0.1
Wrist	1.3 ± 0	1.7 ± 0.6	1 ± 0.3	1.1 ± 0	1.7 ± 1.2	0.7 ± 0.3
L4/L5	2 ± 0.4	2.6 ± 0.1	2.2 ± 0.2	2.1 ± 0.3	3.2 ± 0.7	2.4 ± 0.4
Overall score	50	63	32	39	70	19

**Table 2:** Acronyms for different joint combinations. The names of the rotational axes of the joints correspond to anatomical terms of direction when the wearer is standing upright with both shoulders flexed (90°, 0° abduction). The abbreviation of those axes is reflected in the acronym. An illustration of the joint combination (left: hip belt, right: arm bracing) is presented in the corresponding columns.

Acronym	Hip Connection	Illustr.	Arm Connection	Illustr.
H: R.cc–A: R.lm	Revolute joint with craniocaudal axis	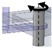	Revolute joint with lateromedial axis	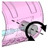
H: R.cc–A: R.cc	Revolute joint with craniocaudal axis	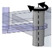	Revolute joint with craniocaudal axis	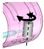
H: R.cc–A: U.lm-cc	Revolute joint with craniocaudal axis	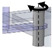	Universal joint with lateromedial and craniocaudal axis	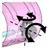
H: U.cc-ap–A: R.lm	Universal joint with craniocaudal and anteroposterior axis	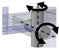	Revolute joint with lateromedial axis	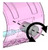
H: U.cc-ap–A: R.cc	Universal joint with craniocaudal and anteroposterior axis	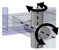	Revolute joint with craniocaudal axis	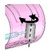
H: U.cc-lm–A: R.cc	Universal joint with craniocaudal and lateromedial axis	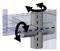	Revolute joint with craniocaudal axis	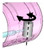

**Table 3:** Average MAs, JFs, parasitic forces (axes: distoproximal, lateromedial), and torques (axes: distoproximal, craniocaudal) over the left and right side of the body and the motion sequence T5 for six exoskeleton concept variants. Variants are combinations of a joint in the row Hip connection and a joint in the row Arm connection, respectively. The acronyms of the joints are shown in Table 2. The last row shows the results of an overall rating of the concepts, with lower numbers indicating better results.

Hip Connection	No Exoskeleton	R.cc			U.cc-ap		U.cc-lm
Arm Connection		R.lm	R.cc	U.lm-cc	R.lm	R.cc	R.cc
Muscle activities MA [%]							
Shoulders	37.1	13.8	15.1	13.1	13.4	26.9	13.2
Back	27.9	8.9	9.2	9.0	9.5	10.3	9.6
Joint forces JF [N]							
Glenohumeral	1495.0	287.2	329.9	319.1	298.4	461.3	334.2
L4/L5	1665.3	401.5	429.8	432.7	448.5	502.1	460.5
Arm bracing forces [N]							
Distoproximal	-	4.2	2.6	10.8	5.5	5.0	4.2
Lateromedial	-	38.1	31.9	37.5	22.7	15.4	39.7
Arm bracing torques [Nm]							
Distoproximal	-	8.0	11.4	6.5	6.3	11.8	7.8
Craniocaudal	-	14.5	4.6	2.5	3.7	4.5	3.0
Overall rating	-	25.0	28.0	22.0	24.0	39.0	30.0

## Data Availability

The data presented in this analysis are available on request from the corresponding author.
